# Supplementary material for: Brain insulin signaling restores deficits in striatal dopamine release in overweight male mice with preexisting low D2-receptor expression
Source: bioRxiv. 2025 Oct 30:2025.10.28.685173. Preprint. [Version 1] doi: 10.1101/2025.10.28.685173 (PMC12636313; doi:10.1101/2025.10.28.685173)
Supplement: Supplement 1 [file media-1.pdf]

EXTENDED DATA- Bocarsly et al. “Brain insulin signaling restores deficits in striatal dopamine release in overweight male mice with preexisting low D2-receptor expression”

Extended Data Figure 1. Bocarsly et al.

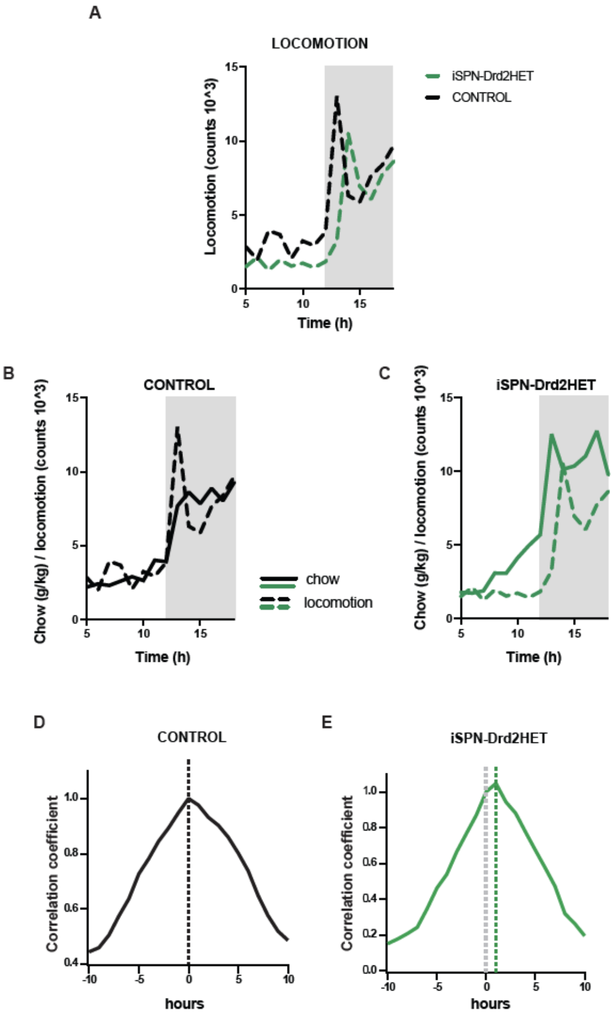

Extended Data Figure 1. Delayed onset of circadian locomotor activation in male mice with low striatal D2 receptors

A. Expanded timeline in the circadian cycle for the 24-h locomotor activity, centered around the light change from inactive (light) to active (dark, shaded) phase for iSPN-Drd2HET (green) and littermate control ($Drd2^{LoxP/wt}$, black) mice. Dashed lines are mean. **B-C**. Expanded timeline in the circadian cycle for the 24-h locomotor activity (dashed line) and chow intake (solid line) for **B**, littermate $Drd2^{LoxP/wt}$ control mice (black) and **C**, iSPN-Drd2HET mice (green). Lines are mean. **D-E** Cross-correlograms between locomotor activity and chow intake across the circadian cycle show **D**, the peak at 0 hours for control mice (black) and E, the peak at 1 hour for iSPN-Drd2HET mice (green), indicative of a time shift between the onset of feeding and locomotion.

Extended Data Figure 2. Bocarsly et al.

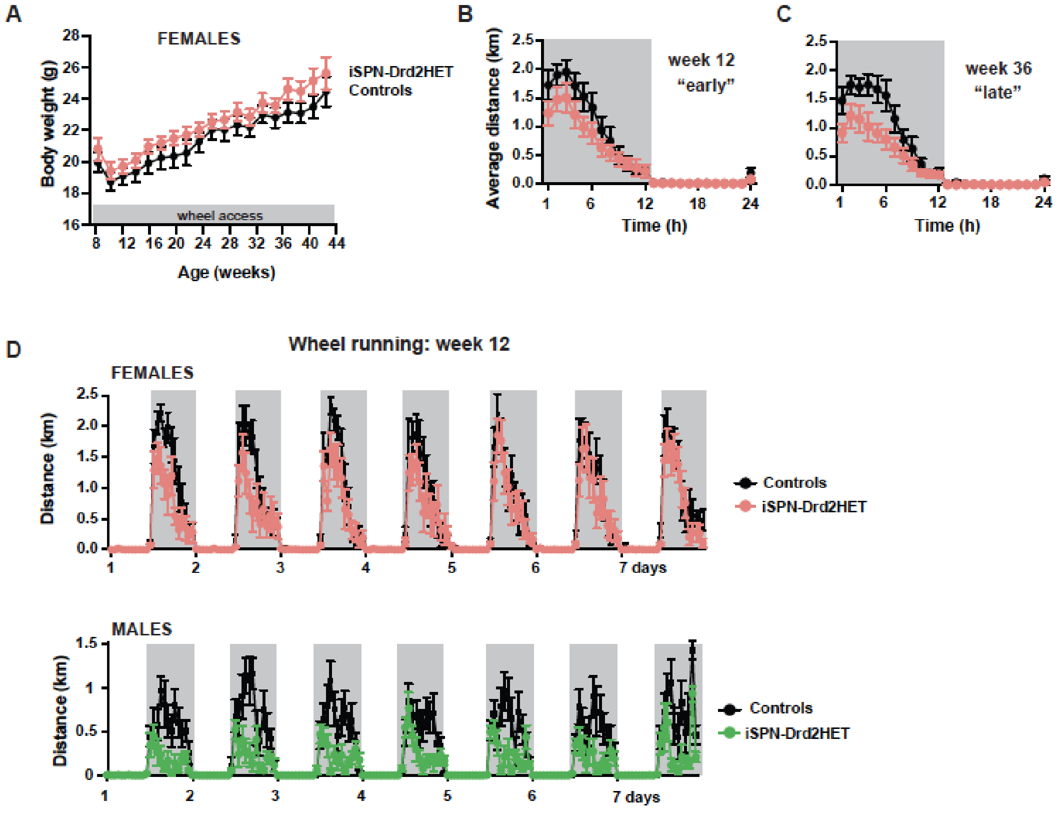

Extended Data Figure 2. Female mice with a low density of D2 receptors in striatal neurons do not show significant differences in body weight and wheel running

A. Longitudinal measure of body weight for female iSPN-Drd2HET (pink) and control (black) mice housed with a running wheel from week 8 - 44. Symbols and lines are mean ± SEM. **B-C**. Running distance per hour across the circadian cycle for iSPN-Drd2HET (pink) and control (black) female mice during (**B**) week 12 and (**C**) week 36. Symbols and lines are mean ± SEM. **D**. Running behavior over a week shows the circadian fluctuations in distance running for (top) female mice littermate Drd2$^{LoxP/wt}$ control (black) and iSPN-Drd2HET (pink) and for (bottom) male mice littermate Drd2$^{LoxP/wt}$ control (black) and iSPN-Drd2HET (green) mice. Symbols and lines are mean ± SEM.

Extended Data Figure 3. Bocarsly et al.

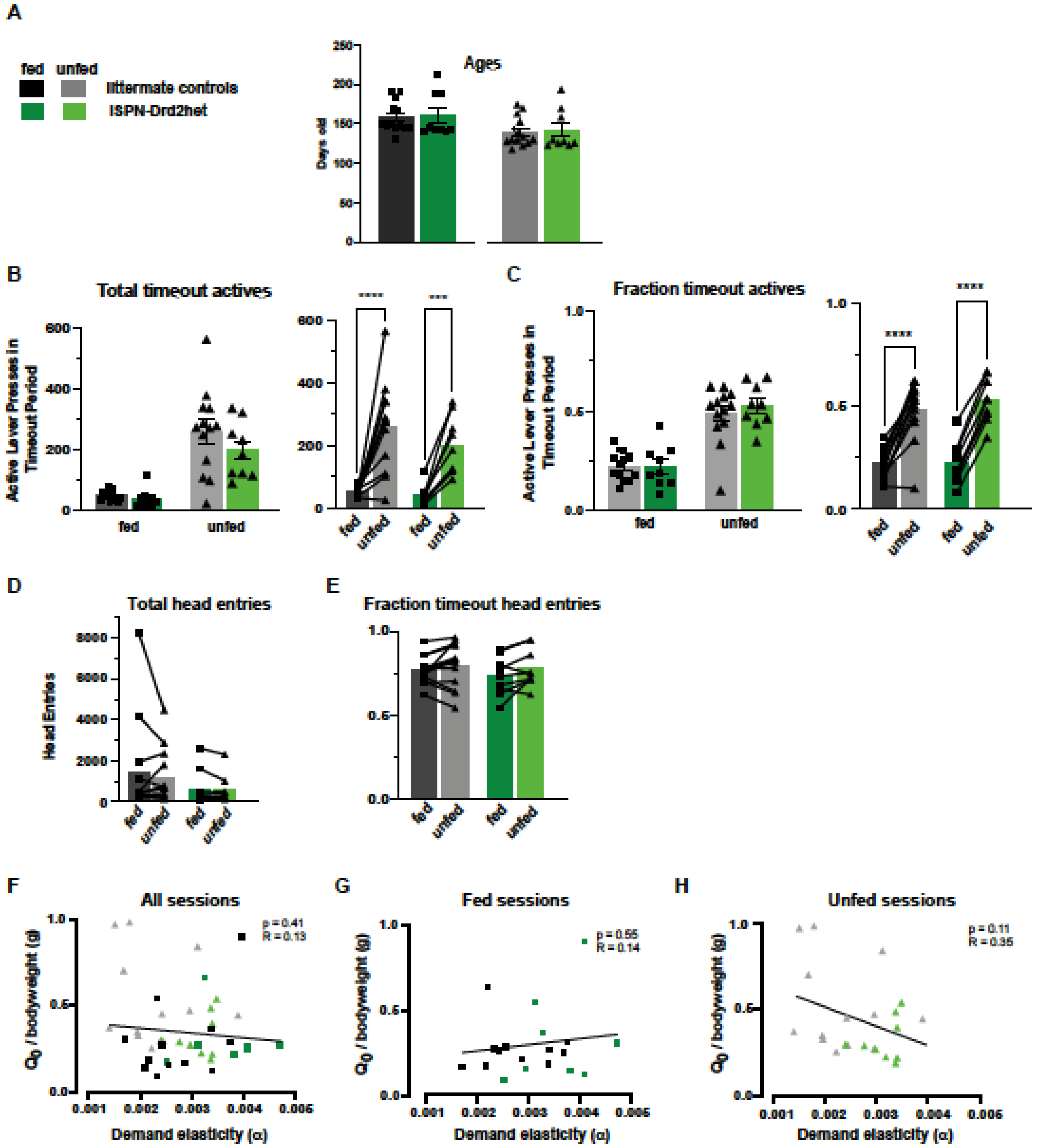

Extended Data Figure 3. No difference in the age or persistent food-seeking behavior across genotypes, and independence of alpha and Q0 parameters

A. Age (in days) of mice on the first day of behavioral economics testing in unfasted and fasted states. **B, C.** Total and fraction of active lever presses in timeout periods within behavioral economics sessions, showing summary (left) and paired (right) data. **D, E.** Total and fraction of timeout head entries within behavioral economics sections, showing paired data within mice. **F, G, H.** Alpha and Q0 fail to show a correlation, indicating the independence of these parameters. Symbols and lines represent mean ± SEM.

Extended Data Figure 4. Bocarsly et al.

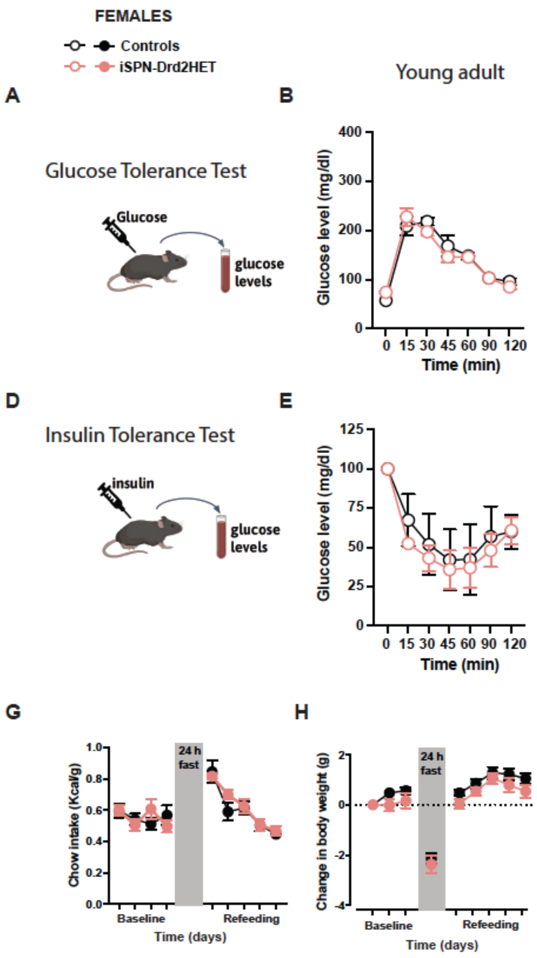

Extended Data Figure 4. Metabolic signs of peripheral insulin resistance in aged overweight female mice

All testing was performed in female young adult (8 weeks, left) and aged adult (60 weeks, right) iSPN-Drd2HET (pink) and control (black) mice. **A.** Schematic of i.p. glucose tolerance test. Mice were fasted overnight, administered an i.p. bolus of glucose, and blood glucose was monitored for 2 hours. **B.** Female mice were tested as young adults and **C.** aged adults. **D.** Similarly, on the i.p. insulin tolerance test, mice were fasted, however, this time they were administered an i.p. bolus of insulin, and blood glucose was monitored for 2 hours. As above, female mice were tested as **E.** young adults and **F.** aged adults. **G.** Chow intake and **H.** bodyweight were measured before and after a 24 h fasting period. No differences were seen between genotypes. For all panels, symbols and lines represent mean ± SEM.

Extended Data Figure 5. Bocarsly et al.

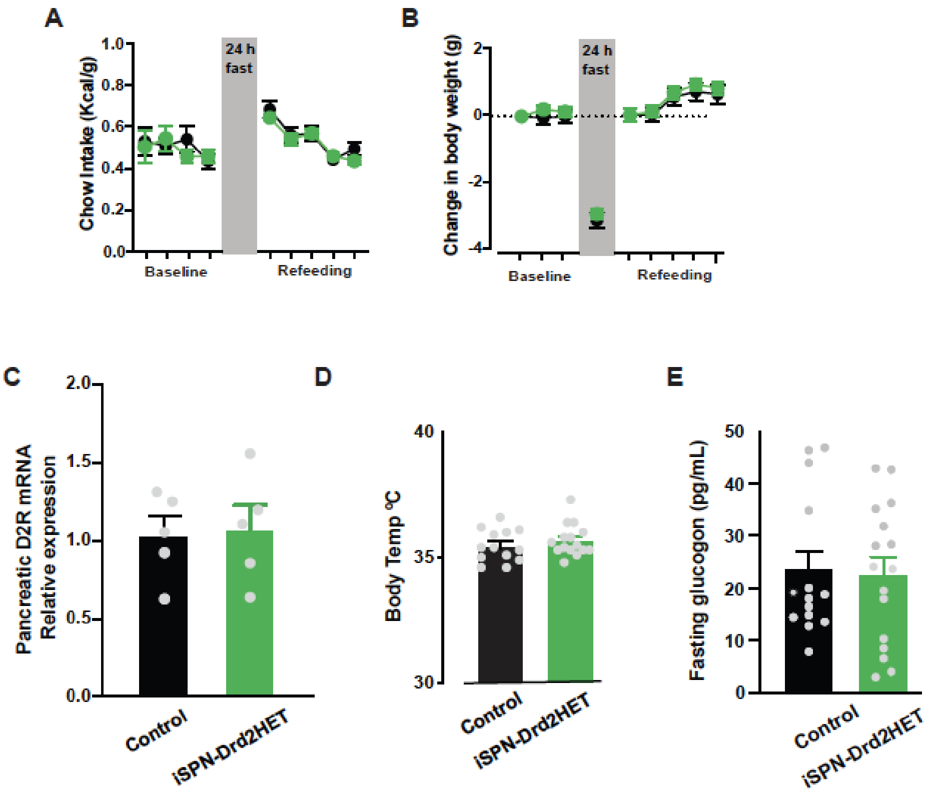

Extended Data Figure 5. Not all peripheral markers of metabolism are changed in overweight male mice.

A. Food intake and **B.** body weight were no different before and after a fasting re-feeding task in adult iSPN-Drd2HET mice and littermate controls. **C.** Drd2 mRNA levels were no different in pancreatic islets in male iSPN-Drd2HET mice and littermate controls. **D.** Basal body temperature was no different between male iSPN-Drd2HET mice and littermate controls. **E.** Fasting blood glucagon levels were no different between male iSPN-Drd2HET mice and littermate controls. All data is mean ± SEM.

Extended Data Figure 6. Bocarsly et al.

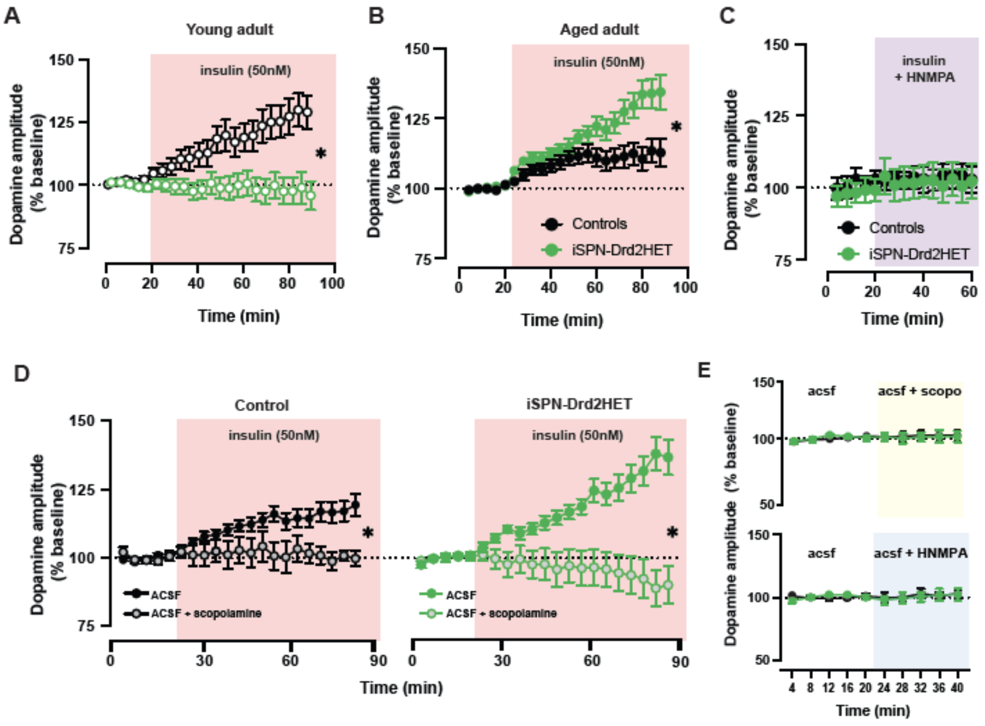

Extended Data Figure 6. Time course of *ex vivo* dopamine response in young and aged male mice with low D2 receptors.

A. In early adulthood (8 weeks) ex vivo FSCV was completed in fasted male iSPN-Drd2HET mice and littermate controls. When slices were treated with 50nM insulin, an increase in evoked dopamine was seen in control mice but absent in mice with low D2 receptors. **B.** When the same experiment was performed using tissue from 60-week-old, aged mice, while controls still showed an increase in evoked dopamine in response to insulin, mice with low D2 receptors showed an enhanced dopamine response to insulin. **C.** Control experiments demonstrate that the insulin receptor blocker, HNMPA, occludes the dopamine response to insulin in both iSPN-Drd2HET mice and littermate controls. **D.** Likewise, bath application of the acetylcholine muscarinic receptor blocker, scopolamine, blocks the dopamine response to insulin in both iSPN-Drd2HET (right) mice and littermate controls (left). **E.** Neither HNMPA nor scopolamine had any effects on evoked dopamine levels in the absence of insulin. Data is presented as mean ± SEM. * denotes statistical significance $p < 0.05$.
